# Clinical research during the COVID-19 pandemic: The role of virtual visits and digital approaches

**DOI:** 10.1017/cts.2021.19

**Published:** 2021-03-08

**Authors:** Tammy L. Loucks, Clare Tyson, David Dorr, Vesna D. Garovic, James Hill, S. David McSwain, Sally Radovick, Frank A. Sonnenberg, Jennifer A. Weis, Kathleen T. Brady

**Affiliations:** 1 South Carolina Clinical and Translational Research (SCTR) Institute, Medical University of South Carolina, Charleston, SC, USA; 2 Department of Medical Informatics and Clinical Epidemiology, Oregon Health and Science University, Portland, OR, USA; 3 Department of Internal Medicine, Division of Nephrology and Hypertension and Department of Obstetrics and Gynecology, Mayo Clinic College of Medicine, Rochester, MN, USA; 4 Center for Clinical and Translational Science (CCATS), Mayo Clinic, Rochester, MN, USA; 5 Department of Nutrition Sciences, University of Alabama Birmingham, Birmingham, AL, USA; 6 Department of Pediatrics, Medical University of South Carolina, Charleston, SC, USA; 7 Department of Pediatrics, Rutgers Robert Wood Johnson Medical School, New Brunswick, NJ, USA; 8 Department of Medicine, Rutgers Robert Wood Johnson Medical School, New Brunswick, NJ, USA; 9 Department of Psychiatry and Behavioral Sciences, Medical University of South Carolina, Charleston, SC, USA

**Keywords:** COVID-19 pandemic, virtual systems, clinical trials

## Abstract

Clinical trials are a fundamental tool in evaluating the safety and efficacy of new drugs, medical devices, and health system interventions. Clinical trial visits generally involve eligibility assessment, enrollment, intervention administration, data collection, and follow-up, with many of these steps performed during face-to-face visits between participants and the investigative team. Social distancing, which emerged as one of the mainstay strategies for reducing the spread of SARS-CoV-2, has presented a challenge to the traditional model of clinical trial conduct, causing many research teams to halt all in-person contacts except for life-saving research. Nonetheless, clinical research has continued during the pandemic because study teams adapted quickly, turning to virtual visits and other similar methods to complete critical research activities. The purpose of this special communication is to document this rapid transition to virtual methodologies at Clinical and Translational Science Awards hubs and highlight important considerations for future development. Looking beyond the pandemic, we envision that a hybrid approach, which implements remote activities when feasible but also maintains in-person activities as necessary, will be adopted more widely for clinical trials. There will always be a need for in-person aspects of clinical research, but future study designs will need to incorporate remote capabilities.

## Overview and Rationale for Change

Clinical trials are a fundamental tool in evaluating the safety and efficacy of new drugs, medical devices, and health system interventions. As such, clinical trials are critical in the development of new therapeutics and improvements in disease prevention and management. Clinical trial visits generally involve participant eligibility assessment, eligible participant enrollment, intervention administration, data collection, and follow-up. Many of the steps in this process are traditionally performed during face-to-face visits between potential and enrolled trial participants and the investigative team. The intensity of physical contact and duration of study visits varies tremendously depending on the intervention under investigation, but at a minimum, study visits often include the collection of vital signs and specimens for laboratory measures.

The coronavirus disease 2019 (COVID-19) has been an impetus for a transformational change in clinical research, as social distancing forced study teams to transition to a virtual format for some key study activities. Many research teams were advised that in-person research would cease for all but life-saving treatment, so initially, screening and enrollment of new study participants was largely halted. However, the needs of participants already actively enrolled in studies could not be ignored. Clinical research teams were challenged to identify novel methods to conduct key aspects of a study (e.g., study medicine dispensing, safety labs, study procedures) that adhered to institutional mandates restricting in-person research, while maintaining regulatory compliance, meeting ethical obligations to participants and the research process, and mitigating financial impacts.

Although the pandemic has sped up the embrace of remote technologies for clinical research, their promise has been recognized for some time. Investigators have been exploring how digital technologies and advances could improve study feasibility and efficiency and lead to more representative study populations [1, 2], as well as inform innovative study designs [3] and streamline clinical trial costs [3]. In the best of times, a number of barriers make it difficult for researchers to recruit participants who reflect the diversity of their communities. Clinical trial participation can be too burdensome and expensive for those who do not live close to a research site or who have mobility or scheduling constraints, increasing disparities in access to research and limiting participant diversity. Provided that caution is taken not to deepen the digital divide, remote and other digital technologies could offer a more patient-centered trial experience, relieving participant burdens and costs related to travel and missed work or school, factors contributing to trial failure [4]. In so doing, they could also pave the way for recruiting more diverse and underserved populations into clinical trials.

The purpose of this special communication is to describe the advancement of virtual visits and related methods for several critical clinical research activities and highlight important considerations associated with virtual approaches that represent areas for future development in moving clinical research to a decentralized or hybrid model. This work was informed both by responses obtained from a CTSA-wide survey and by the experiences of the authors in navigating the aforementioned challenges in their own research programs.

### CTSA Survey

**Fig. 1.**
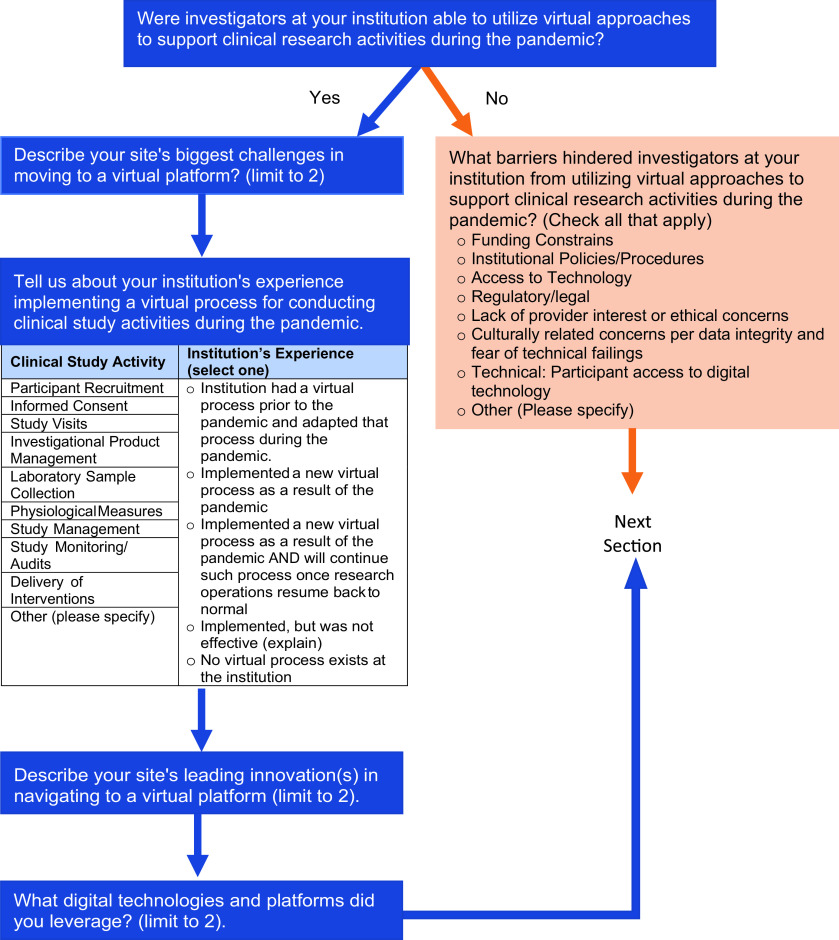
Depiction of the portion of the CTSA COVID-19 survey related to virtual visits.

**Fig. 2.**
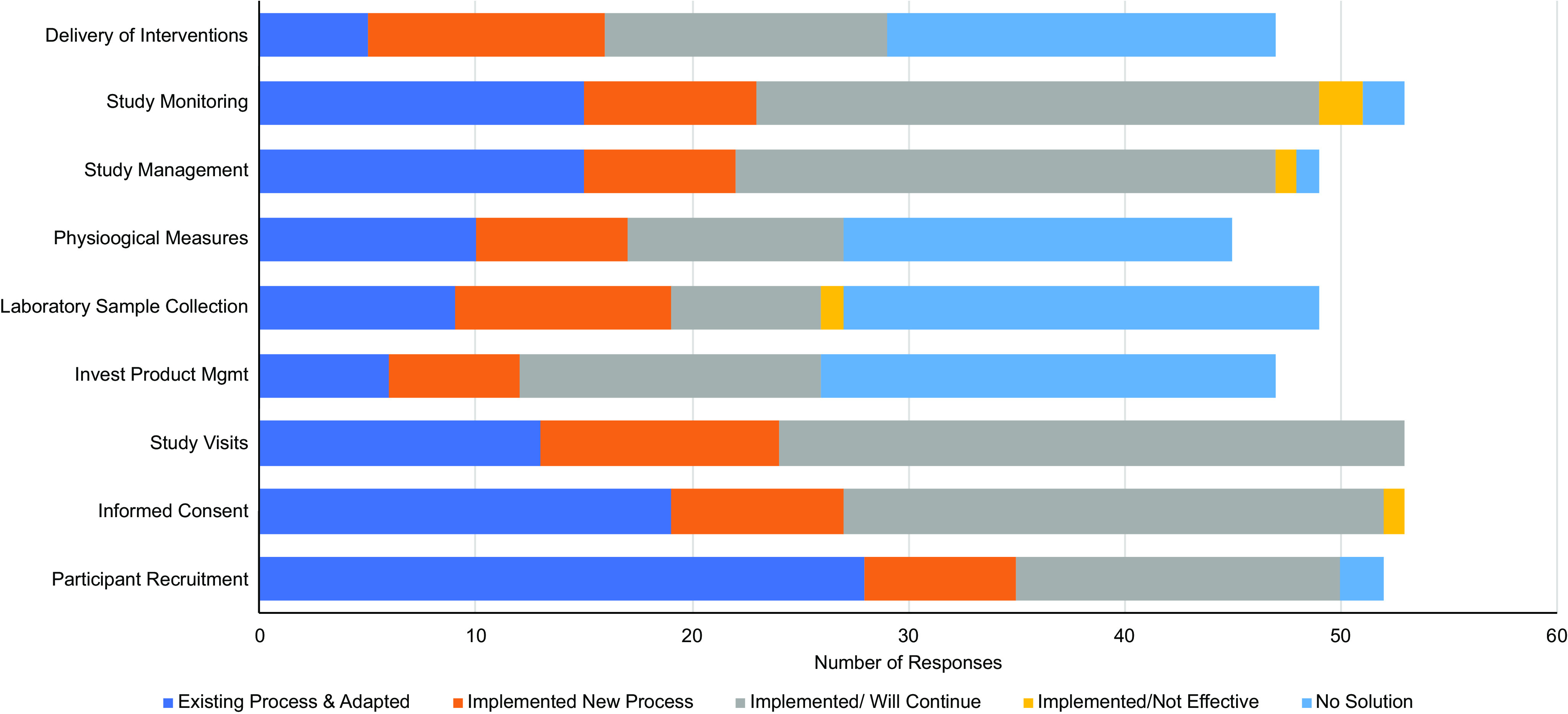
Institutional experiences implementing a virtual process for conducting clinical study activities during the pandemic.

The Center for Leading Innovation and Collaboration (CLIC) survey team was enlisted to develop a CTSA-wide survey to gather information about innovations and adaptations made by hubs in response to the COVID-19 pandemic. This survey would be used to provide information to writing groups preparing special communications about informatics, biorepositories, virtual visits, institutional review board (IRB), protecting research personnel, prioritizing COVID-19 studies, COVID-19 adaptations to pharmacy procedures, and prioritizing non-COVID-19 studies. CLIC survey team members met with writing groups to review and restructure their proposed questions when appropriate. Hub principal investigators (PIs) were asked to complete one REDCap survey per hub by October 15, 2020. PIs were also encouraged to solicit responses from others at their hub to gather expert-specific data using a Word document version of the online REDCap survey that was provided. The portion of the survey related to virtual research visits consisted of six questions (Fig. 1). Of the 65 hubs that received the survey, 60 responses were gathered for a response rate of 92.3%. Eighty-two percent of hubs reported disruptions in their research operations beginning between January and March of 2020. Fifty-eight of the sixty programs provided responses to the survey questions about virtual visits. Of these programs, 56 (97%) reported that a virtual approach was available for some aspect of their clinical research. Two programs (3%) reported that they did not adopt virtual approaches due to a number of barriers, including lack of access to technology for participants and study teams, fiscal limitations, institutional policies, and/or regulatory constraints. The types of research activities conducted virtually, as well as the processes associated with the availability of these methods to study teams, are depicted in Fig. 2. These activities can be classified as those involving enrollment, study coordination and conduct, and regulatory issues and compliance audits.

### Enrollment

#### Recruitment and Screening

For research activities deemed vital to continue during the pandemic, it was necessary to determine if in-person visits were necessary or if activities could be conducted in part, or in full, through virtual visits. Research necessitating in-person visits required careful consideration of the potential benefits a therapeutic intervention might offer, compared to the health risks of possible exposure to COVID-19.

Research activities conducted via remote visits offered trial participation without the associated risk of potential exposure to COVID-19. Researchers at CTSA hubs across the nation began to employ a host of existing but infrequently utilized remote options to facilitate ongoing trial recruitment, screening, and enrollment. According to the CTSA survey (Fig. 2), most programs were able to use an existing process or implement a new one to enable virtual visits for participant screening and enrollment. Both phone and video visits were employed in place of in-person recruitment and screening visits. The transition of research activities from in-person to virtual required close coordination with study sponsors and with IRBs, which had to approve protocol amendments. Screening visits transitioned to existing telehealth platforms, with Doxy.me, WebEx, Zoom, and FaceTime examples provided by respondents.

#### Informed Consent

The Food and Drug Administration (FDA) issued guidance (with subsequent updates) on conducting clinical trials during the pandemic that included a question-and-answer appendix [5]. Aiming to mitigate risk during the pandemic while ensuring compliance with Good Clinical Practice (GCP), the FDA provided flexibility to sites for obtaining informed consent when electronic methods were not available. This document also included recommendations for obtaining consent from quarantined participants. The guidance, however, did not provide much flexibility to FDA-regulated studies with respect to 21 CFR Part 11 and electronic signatures, which remains a significant challenge to rapid implementation at a site level.

Our survey indicated that some CTSA institutions did have Part 11 electronic signature capabilities supporting the continued use of paper–pen to obtain consent via telehealth platforms. Documents were routed to participants in advance of their remote visits using various digital platforms that allowed for electronic review and digital signature by the participant (i.e., DocuSign, PTrax). In addition, consent documents were also e-mailed directly to participants for review and consideration. Because e-mail does not allow for digital signatures, recipients had to print and sign the consent form and then send a photo of the signed consent to the study team. The team would then print and sign the document and upload it into the electronic health record (EHR).

Many CTSA sites were able to offer virtual informed consent by quickly transitioning from paper–pen informed consent to established HIPAA-compliant eConsent platforms, such as Doxy.me and REDCap. Our survey indicated that 12 CTSAs (20%) used REDCap eConsent, 11 (18%) referenced using REDCap but did not specify the eConsent module, and 9 (15%) referenced moving to eConsent but did not identify the specific platform used. Vanderbilt University shared its REDCap eConsent validation materials with other CTSAs for a more expeditious implementation of a Part 11-compliant eConsent platform [6]. For sites unable to validate systems for Part 11 compliance quickly, study sponsors were able to provide other eConsent platforms. The FDA also released the COVID-19 MyStudies App at no cost for eligible studies. These systems still required vetting through the institution’s information technology and cybersecurity offices and review by the IRB.

Importantly, the flexibilities enabled by the FDA under the Public Health Emergency declaration are not permanent. Once the pandemic is over, virtual consent approaches will be important considerations to include during the study design phase.

### Study Coordination and Conduct

#### Assessments and Biomarkers

As states implemented stay-at-home orders, research teams and sponsors were faced with determining which safety and primary outcome measures were essential and which could be missed or postponed. Study teams and sponsors considered alternatives to previously designed face-to-face visits, citing FDA regulations that allowed for “exceptions where necessary to eliminate apparent immediate hazards to the human subjects” (21 CFR 56.108(a) [5]. Research teams at many sites swiftly partnered with sponsors to establish study-specific plans that protected the safety and welfare of participants without compromising study integrity. Temporary use of virtual visits, whether by telephone or telemedicine, was instituted.

Telemedicine platforms enabled teams to pivot quickly to collect virtual assessments and maintain contact with study participants [7]. Most participants viewed the opportunity for virtual study visits very favorably, as is consistent with previous findings [8], noting the convenience, ease of access, and lack of exposure during the pandemic. Many researchers were already familiar with delivering remote assessments via survey platforms such as REDCap, Qualtrics, and Survey Monkey [2], and some had already implemented self-report assessments before the pandemic via virtual formats such as electronic patient-reported outcomes (ePROs), eDiary’s, and HUGO. In addition, many study teams at CTSA hubs were already using wearable devices to capture participant data, including vital signs, physical activity, sleep patterns, and even falls [9].

At the height of the pandemic, the immediate challenge to implementing electronic capture or wearable devices was that they were not part of the original study design. Another issue was that digital health technologies, though widely employed for personal use, had yet to be integrated routinely into clinical research as many still required validation [1]. Other challenges included the inability of some patients to complete remote assessments due to lack of familiarity with or limited access to digital portals on their personal devices. To address this barrier, many research teams collected study participant information through telehealth and manually entered the assessment data into case report forms (CRFs). Other study teams generated REDCap and Qualtrics surveys that could be e-mailed to participants for completion.

When virtual methods were deemed insufficient for certain trials, study teams relied on local providers to conduct assessments and obtain biometric measures such as vital signs, height, weight, blood pressure, temperature, pulse, and respiratory rate. Their involvement, like the use of wearable devices to capture biometric data, required prior sponsor and IRB approvals. Permission was also required before information collected by local providers could be released and shared. All data obtained from local providers had to be entered into the CRFs by the enrolling site. In some situations, the sponsor permitted the site to skip a full assessment of vital signs provided that a remote clinical examination had taken place and a deviation had been submitted to this effect. Despite close coordination with local healthcare providers, the investigator enrolling the participant retained responsibility for participant safety, data management, study reporting, review of lab and image results, and tracking and reporting of adverse effects.

The broad use of EHRs greatly facilitated the sharing of data among study teams, local providers, and patients. Data could either be directly uploaded through existing platforms (e.g., Epic Care Everywhere), or participants could access and forward their own medical records for review and consideration.

Despite their challenges, virtual methodologies are likely here to stay in clinical research. Indeed, remote patient assessment and electronic capture of biomarker data using devices, smartphones, and sensors will likely be a cornerstone of a decentralized model of clinical research. These biomarker technologies could provide researchers with real-time data between research visits, resulting in earlier detection of adverse and safety events [1]. Before their potential can be realized, however, a number of issues will have to be addressed. The pandemic revealed a need for better validation of devices and for the ability to compare and integrate data collected across all devices. Data security, privacy, and participant trust issues must also be addressed. Increased partnership with regulators will be required to reduce the risk of potential exploitation as these digital tools gain momentum [10]. The pandemic will likely act as an accelerant to these efforts, speeding the adoption of such digital measures in clinical research.

#### Laboratory Issues

Management of screening results required close coordination with study sponsors, who approved a variety of methods for collecting study samples. Some sites deployed local home health agencies to collect blood, urine, and/or stool samples from patients and send them by courier to the participating sites for processing. Sponsors also approved the shipment of lab kits directly to participants for sample collection at a nonparticipating local laboratory. In some cases, the local laboratory sent the collected samples to a central lab for processing, while in others, it performed the study assays itself. Once participant approval was obtained, lab results were shared with the participating study site. Study staff ensured that lab results were within established trial parameters and recorded lab results in the studies’ CRFs. In some instances, sites were permitted to forego certain labs provided that participants had been stable on their medications for some time and ongoing safety lab results remained within the normal range. This flexibility was especially important when virtual options for laboratory sample collection were not available, as was true for 45% of programs responding to our survey (Fig. 2). Similarly, participants were permitted to undergo imaging at a local facility as long as the study team was able to review the imaging remotely. Thanks to these varied collection options, study teams were able to offer continued safety monitoring during the pandemic.

#### Drug Management

Study drug management was a challenge to which a number of CTSA sites had no virtual solution (Fig. 2). Instead, a variety of approaches were taken, including personal delivery of study drugs to participants by research staff, use of medical delivery services (e.g., MedSpeed), curbside or valet medication pickup, shipping of study medications through the mail, and use of home health for parenteral medications. All alternative methods to deliver study agents required prior IRB approval.

Investigators and study sponsors also had to consider the possible impacts of alternative arrangements on trial participants, in some cases with limited ability to monitor participants closely. Another concern was modifying trials that offered potential benefits to participants who were more concerned about managing their existing health issues than contracting COVID-19. In all cases, sites diligently informed trial participants of study changes and their potential impacts as well as the need for close follow-up.

#### Study Remuneration

Study visit remuneration, in general, remained the same regardless of whether a visit was conducted virtually or in person. Many sites, which were already using checks and retailer e-gift cards for patient remuneration, simply began to mail them out during the pandemic. Several CTSA hubs were already using Greenphire’s ClinCard, a secure online participant payment system that allows sites to track and make secure payments quickly and easily after each study visit. As sites changed to a virtual platform, W9 information, previously captured on paper, began to be obtained by and stored in secure online tools, such as REDCap. Studies that transitioned to different methods of remuneration due to the pandemic required IRB modifications to the informed consents, as well as IRB approval prior to implementation.

### Sponsor Input

#### Source Documentation

Despite the FDA’s guidance on the use of electronic sources [11], clinical research has been slow to transition to electronic source documentation, continuing to rely heavily on paper. With the pandemic, research teams using paper source documentation needed to pivot quickly to electronic data capture and storage of source documentation to manage study operations remotely. Teams had to evaluate secure and HIPAA-compliant options to collect, store, manage, de-identify, and review documentation that was consistent with local, institutional, and sponsor requirements. Additionally, for FDA-regulated studies, teams needed solutions that would enable study monitor access for review and verification of source documentation and sponsor-endorsed methods for obtaining electronic signatures. Many sites used existing institutionally approved technology systems to make this initial transition.

Sites collaborated with sponsors to identify editable electronic documents (e.g., fillable PDFs, Word documents) for assessments or other source data and a mechanism for storing these documents within HIPAA-compliant and institutionally supported secure systems, such as Box, Microsoft OneDrive, SharePoint, and Filelocker. Electronic source documentation was facilitated when study sites used the EHR to document research-specific procedures and assessments (e.g., research note, adverse event logs, informed consent), performed direct data entry into electronic CRFs (eCRFs), relied on ePROs, or used REDCap and Clinical Trial Management Systems to maintain essential files and regulatory documents.

The urgency to transition immediately to electronic sources may have led sites to implement a provisionary solution during the initial stages of the pandemic. However, as research shifts to a decentralized model, it is clear that electronic source data will need to be further explored. The pandemic has provided a tremendous opportunity to streamline and replace some of the antiquated research processes and to use electronic source documentation as a more effective and efficient way to capture and monitor clinical trials data in real time [12].

#### Study Monitoring

Across all aspects of study conduct, the transition to clinical trial remote monitoring was most seamlessly facilitated at sites using Epic as their EHR through the EpicCare Link feature, which is 21 CFR Part 11 compliant. Requests for a remote monitoring visit corresponded with the study timeline and duration delineated in the study agreement and all confidentiality and contractual agreements were applied to remote study monitoring activities. Once their EpicCare Link privileges were approved, study monitors could access identified participants’ records remotely. As with in-person monitoring, site study staff needed to be available to address questions or to produce requested documents, such as source documentation not housed in the EHR, and to provide those documents using a secure file-sharing solution, such as Simple Share. Virtual video meetings were implemented for site initiation and qualification visits. Anecdotally, many sponsors have reported that remote monitoring visits have been very successful and have led to increased efficiencies. They plan to encourage participating sites to continue using remote processes following the pandemic.

### Regulatory Issues and Compliance Audits

Two of the CTSA survey sites identified institutional policy or regulatory legal issues as barriers to using virtual approaches. Indeed, there are significant regulatory concerns associated with virtual visits, including the requirement to develop informed consent processes for data collection, transmission, and sharing; endpoint validation for claims and EHR data, as well as data obtained from digital health technologies; and strategies for addressing the security, privacy, and ethics concerns raised by the selected digital platform. Implementing virtual technologies can be particularly difficult for multisite clinical trials because regulations regarding telehealth licensure differ by state.

To ease the transition of current clinical trials to virtual platforms, both the FDA [5] and United Kingdom’s Medicines and Healthcare Products Regulatory Agency (MHRA) [13] have released guidance that allows some regulatory flexibility during the COVID-19 crisis. However, as virtual visits are relatively new, institutions have been somewhat slow to adopt these changes. Sponsors are also hesitant to risk adopting this relatively unfamiliar process when considering the cost of instituting and conducting a clinical trial. Greater acceptance of virtual technologies will hinge on heightened security measures for remote monitoring to protect against data breaches during collection, transmission, and/or storage of data. These include risks to confidentiality for trial participants, including their GPS/location data, which may be used for legal actions and incur an economic loss due to stigma. It is unlikely that the aforementioned HIPAA enforcement discretion regarding the use of noncompliant videoconferencing platforms in health care [14] will be continued beyond the public health emergency, further emphasizing the need to address privacy concerns as we move toward a more digitally enabled research environment.

Current FDA regulations address guidelines for personnel oversight responsibilities but do not focus on encounters with study participants. Some regulatory questions remain unanswered despite the regulatory agencies’ interest in working collaboratively to implement new virtual platforms for clinical trials. As a result, virtual trials are currently following variable guidelines issued by individual institutions. Regulatory uncertainty associated with the lack of standardized guidelines may be the reason why some CTSA institutions were unable to conduct virtual trials. However, regulatory agencies, institutions, and sponsors all agree that standardized policies and regulations addressing data integrity and participant safety and privacy are needed.

### Resource Considerations

During the pandemic, more than half of patient encounters at many institutions took place by phone or telemedicine [15]. Likewise, clinical research had to grow its capacity for large-scale virtual visits rapidly, requiring researchers to adapt to new ways of communicating with participants and new technologies for conducting clinical trials.

#### Communications

For health systems, the overall shift to virtual visits necessitated greater use of communication technologies and exacerbated the digital divide, with Black and Hispanic populations seeking virtual care at much lower rates [16]. The CTSA survey showed that communications also presented a challenge to researchers navigating the transition to virtual study visits. Indeed, researchers at 22 of the 56 institutions reported that access to participants was a significant barrier to adopting virtual approaches. Clinical researchers, who are accustomed to relying on multi-modal approaches to recruit patients, found themselves facing challenges on every front. Many researchers were working at home, necessitating that they learn new ways of recruiting and engaging participants. Participants also had to master new communications technologies and, although data are more limited, some of those also fell victim to the digital divide. Finally, researchers had less opportunity to communicate with participants during non-study healthcare visits due to the lack of in-person care and a general decline in overall visits caused by the pandemic. Researchers also reported substantial gaps in their knowledge of and access to systems that would meet the security and privacy requirements for virtual research visits.

#### Videoconferencing

A secure, encrypted, and configurable videoconferencing system is needed for study visits. Early concerns about security and increased load made the selection of these systems challenging. By issuing guidance on enforcement discretion of HIPAA penalties during the public health emergency, The Office of Civil Rights provided the flexibility needed for study teams to use readily available technologies such as FaceTime, Skype, and Google Hangouts when HIPAA-compliant platforms were not available [14, 17]. However, operational use of the video conference systems was often prioritized, leaving researchers challenged to configure systems for their particular needs, such as the transmission of documents and other data.

#### Network Access

In the CTSA survey, many researchers noted challenges in gaining access to virtual private networks (VPNs). They need such access because data collection instruments often require remote desktop capabilities. In cases where VPNs were not available or practical, other means had to be found to ensure secure, encrypted access by both participants and researchers to software needed to complete electronic data collection, monitor study data for quality purposes, and run analyses. Many sites were not set up to access the needed software from home, leading to challenges in completing visits.

### Lessons Learned

**Fig. 3.**
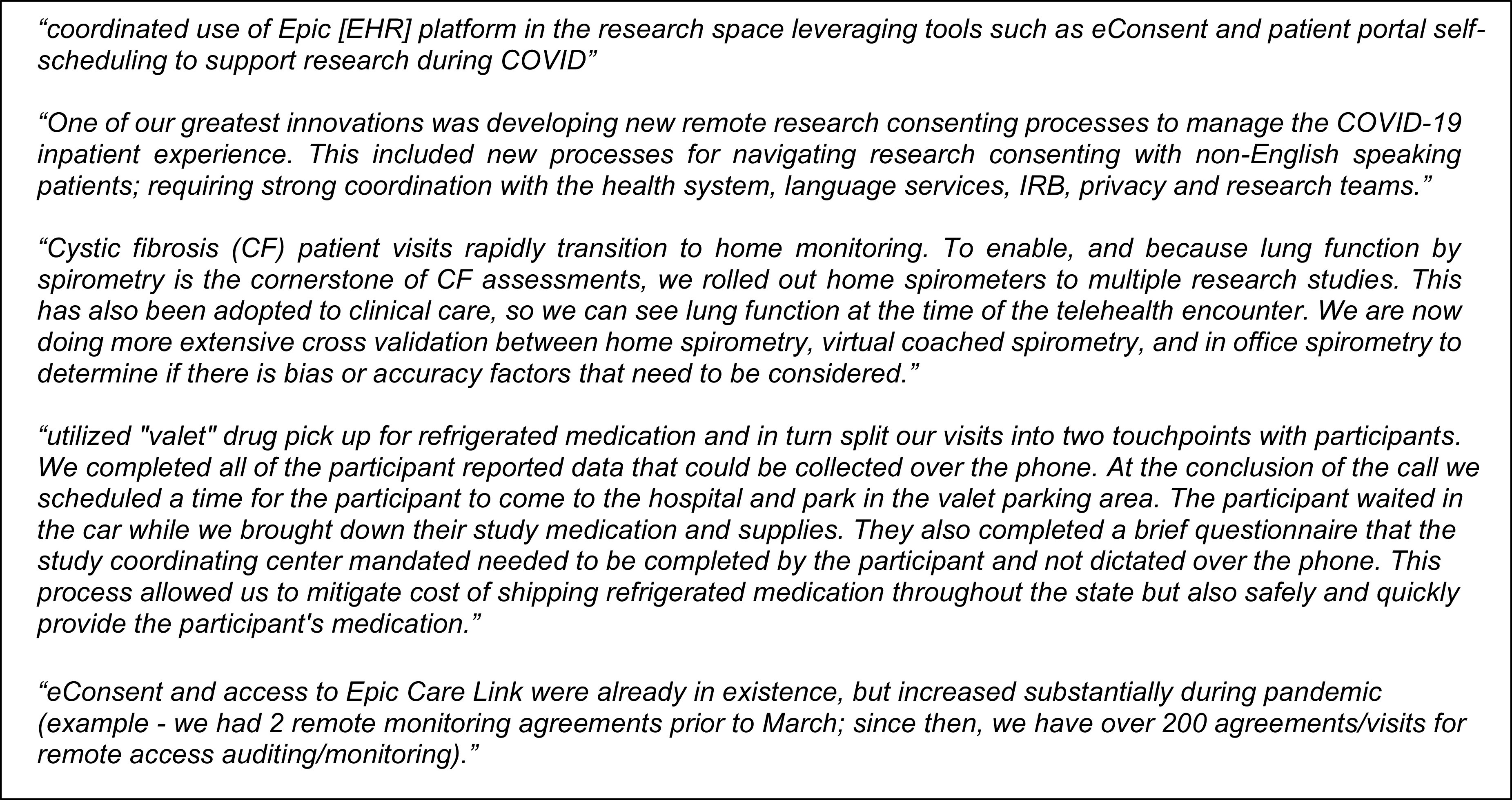
Selected innovations reported by CTSA to enable virtual clinical research visits reported by CTSA programs.

The CTSA survey showed that COVID-19 has disrupted the status quo of clinical research, acting as a catalyst to hasten the adoption of technology-driven solutions as new standards for the field, organizations, and study teams (see comments in Fig. 3). Most programs reported either adapting clinical platforms or implementing new virtual approaches to support research activities, measures that they intend to continue after the pandemic.

Survey responses indicated that a successful transition to virtual options required coordination and collaboration across a broad stakeholder community. This need has also been acknowledged in position statements by experts in cardiovascular disease [18] and oncology [19]. Clinical research owes its rapid adoption of virtual and remote approaches during the pandemic to innovation and new and strengthened interactions among study teams, sponsors, research participants, health systems, informaticians, regulatory officers, and policy-makers, among others. These relationships will be important to maintain after the pandemic if we are to sustain and further develop technological solutions for efficient and high-quality conduct of clinical research.

A few CTSA hubs reported difficulties obtaining laboratory and physiologic measures and delivering an investigational product to participants. In some cases, virtual solutions existed but were not available to study teams because their institutions had not adopted eSignature platforms that were 21CFR Part 11 compliant for source documentation and informed consent.

**Fig. 4.**
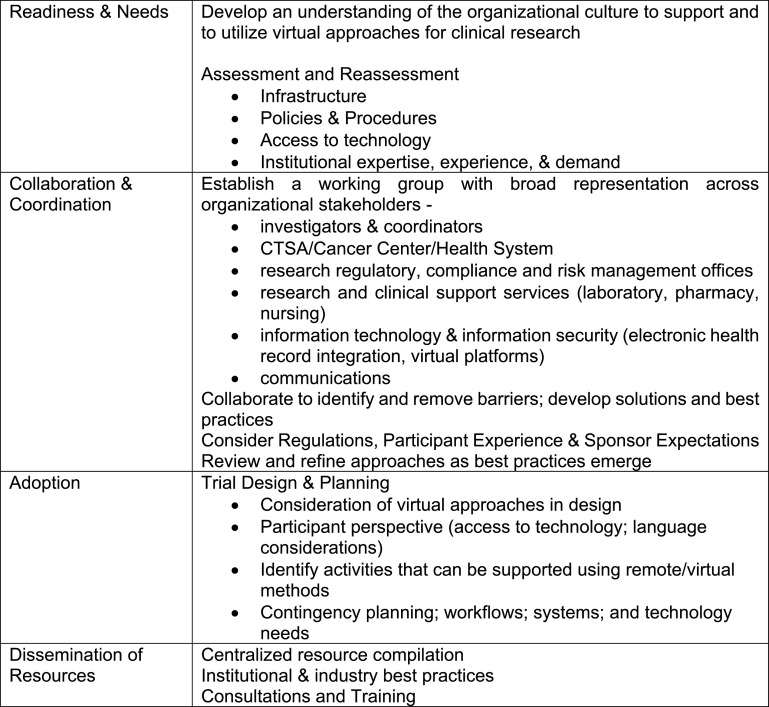
Virtual clinical research playbook.

The collective experience of the CTSA consortium suggests four best practices for academic health centers to ensure a successful transition to virtual clinical research (Fig. 4). These include completing a readiness evaluation, taking a coordinated and collaborative approach across a broad range of stakeholders, integrating technology considerations into trial design and planning, and making virtual resources visible and available to study teams.

### Conclusions

The COVID-19 pandemic served as an impetus for taking advantage of remote capabilities for clinical research. Conceivably, remote research capability could reduce the travel burden on research participants and make it more likely that residents in rural or underserved areas and people with disabilities will have the opportunity to enroll in clinical trials. This could improve the generalizability of trial results, improve efficiency and drive down costs. The issue of participant access, identified by many CTSA programs as a primary barrier, requires thoughtful consideration. Special care needs to be taken to protect against exacerbating the digital divide.

A “silver lining” of the COVID-19 pandemic may be an improved ability to connect with and recruit diverse participants and to deliver therapies and collect data remotely. Ultimately, the best approach may be a hybrid one that implements remote activities when feasible while maintaining in-person activities as necessary. Although clinical research will likely always require some in-person encounters, the pandemic has taught us the importance of incorporating remote capabilities into future study designs.
